# Differential expression of key subunits of SWI/SNF chromatin remodeling complexes in porcine embryos derived in vitro or in vivo

**DOI:** 10.1002/mrd.22922

**Published:** 2017-11-06

**Authors:** Birgit Cabot, Yu‐Chun Tseng, Jennifer S. Crodian, Ryan Cabot

**Affiliations:** ^1^ Department of Animal Sciences Purdue University West Lafayette Indiana

**Keywords:** ARID, BAF155/170, epigenetics, preimplantation embryo

## Abstract

In vitro embryo production is an established method for both humans and animals, but is fraught with inferior development and health issues in offspring born after in vitro fertilization procedures. Analysis of epigenetic changes caused by exposure to in vitro conditions should shed light on potential sources of these phenotypes. Using immunocytochemistry, we investigated the localization and relative abundance of components associated with the SWI/SNF (Switch/Sucrose non‐fermentable) chromatin‐remodeling complex—including BAF155, BAF170, BAF180, BAF53A, BAF57, BAF60A, BAF45D, ARID1A, ARID1B, ARID2, SNF5, and BRD7—in oocytes and in in vitro‐produced and in vivo‐derived porcine embryos. Differences in the localization of BAF155, BAF170, BAF60A, and ARID1B among these sources indicate that improper timing of chromatin remodeling and cellular differentiation might occur in early preimplantation embryos produced and cultured in vitro.

**Abbreviations**
ARID: AT‐rich interaction domain
BAF: Brahma‐associated factor
SWI/SNF: switch/sucrose non‐fermentable

## 1 INTRODUCTION

Chromatin remodeling is a key component of successful development, propagation, and maintenance of mammalian cells and tissues. Epigenetic changes are caused by both covalent modifications of histone proteins e.g., methylation, acetylation, phosphorylation (Endo, Imai, Shimaoka, Kano, & Naito, 2011; Park, Johnson, et al., 2010; Park, Johnson, Wang, & Cabot, 2011; Park, Magnani, & Cabot, 2009; Sun et al., 2002) and non‐covalent modifications of chromatin, which affect gene transcription. Altered transcription profiles were reported in vitro‐produced porcine embryos compared to their in vivo‐produced counterparts (Østrup et al., 2013; Hamm, Tessanne, Murphy, & Prather, 2014), implying that these two sources of embryos affect the embryonic epigenetics. Here, we analyzed non‐covalent chromatin remodeling mediated by SWI/SNF (SWI/SNF, Switch/Sucrose non‐fermentable) chromatin remodeling complexes, which utilize energy from ATP‐hydrolysis to translocate nucleosomes along the chromatin, to understand which of these factors might be affected by in vitro embryo production.

ATP‐dependent SWI/SNF chromatin‐remodeling complexes (also known as Brahma or Brahma‐related gene 1 [BRG1]‐associated factor [BAF] complexes) are multi‐protein complexes that reposition nucleosomes and alter the accessibility of transcription factors to chromatin. Common to all mammalian SWI/SNF complexes is a catalytic subunit that functions as an ATPase (either Brahma or BRG1). In addition, SWI/SNF complexes contain a core group of modulating subunits (BAF150/BAF170 and SNF5) and a multitude of accessory subunits (Euskirchen, Auerbach, & Snyder, 2012; Ryme, Asp, Böhm, Cavellán, & Farrants, 2009); these subunits are encoded by at least 29 genes belonging to 15 gene families (Kadoch & Crabtree, 2015).

Previous studies characterized multiple SWI/SNF complexes in various tissues and cell types (Kadoch et al., 2013). Some of these complexes were found to be involved in tumor suppression, whereby mutations and/or the loss of function of different SWI/SNF subunits are linked to a number of different cancers (Kadoch & Crabtree, 2015; Marquez‐Vilendrer, Rai, Gramling, Lu, & Reisman, 2016; Reisman, Glaros, & Thompson, 2009). BRG1 is expressed in preimplantation embryos, while BRM appears to be expressed later during differentiation (Ryme et al., 2009). Knockout studies in mice identified requirements for these two SWI/SNF subunits that are consistent with their expression during embryo development: *Brg1*‐null mice do not survive beyond early embryonic stages whereas *Brm*‐null mice survive to adulthood and display only a slight overgrowth phenotype. Knockdown of BRG1 resulted in aberrant *Pou5f1* (also known as *Oct4*) and *Nanog* expression in blastocyst‐stage mouse embryos (Kidder, Plamer, & Knott, 2009). This phenotype is likely due to the BRG1 occupancy of the promoters of *Pou5f1, Sox2, Nanog*, and other significant pluripotency related genes, which supports a key role of this factor in the regulation of pluripotency and self‐renewal in embryonic stem cells.

Additional genetic evidence revealed roles of other SWI/SNF factors during development. *Baf155^+/−^* mice develop to term and are fertile, but brain defects (exencephaly) in adults were observed. In contrast, *Baf155* ^−/−^ mice develop to the blastocyst stage, but the inner cell mass subsequently degenerates and fail to develop egg cylinders (Kim et al., 2001). Consistent with these null‐phenotypes, BAF155 knockdown in mice results in aberrant expression of pluripotency genes while overexpression of BAF155 arrested development at the blastocyst stage (Panamarova et al., 2016). Furthermore, gene inactivation of S*nf5* or *Baf155* causes peri‐implantation lethality; *Baf180*‐null mice are lethal at embryonic Days 12.2–15.5, and show cardiac and placenta abnormalities; and murine embryos lacking ARID1A (AT‐rich interaction domain protein 1A) arrest their development around embryonic Day 6.5, with failed development of a mesodermal layer (reviewed by de la Serna, Ohkawa, & Imbalzano, 2006; Gao et al., 2008; Xu, Flowers, & Moran, 2012).

Data regarding SWI/SNF complexes are limited for preimplantation embryos. Results obtained in cell culture and embryonic stem cells, however, provide a baseline understanding of the distinct SWI/SNF complexes present during critical stages of development and differentiation. We hypothesized that SWI/SNF‐complexes in the early embryo should function similarly to SWI/SNF complexes found in pluripotent embryonic stem cells, which are derived from the inner cell mass in mice. We therefore analyzed the localization and relative abundance of a multitude of SWI/SNF subunits during early porcine embryo development, and compared their localization between embryos produced in vitro and those derived from insemination in vivo. We expected to identify changes in the localization and abundance of critical subunits around the time of zygotic gene activation, which occurs at the 4‐cell stage in the pig, and/or morphological differentiation, such as blastocyst formation.

## 2 RESULTS

**Figure 1:**
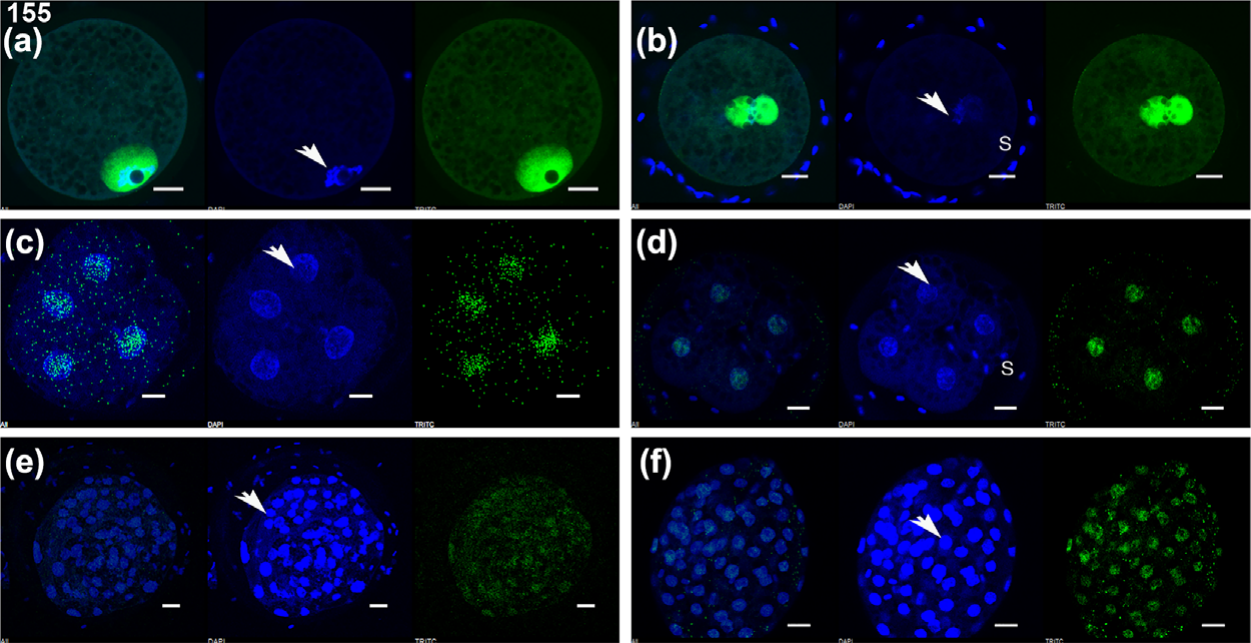
Localization of BAF155 in porcine oocytes and embryos produced in vitro or in vivo. (a) Germinal vesicle‐stage oocyte. (b) Pronuclear embryo produced in vitro. (c) 4‐cell embryo produced in vitro. (d) 4‐cell embryo derived in vivo. (e) Blastocyst‐stage embryo produced in vitro. (f) Blastocyst‐stage embryo derived in vivo. Each panel shows merged images of DNA (blue) and SWI/SNF subunit (pseudo‐colored green) staining (left), or individual DNA (middle) or subunit (right) staining. Arrow, nucleus; S, sperm. Scale bars, 25 μm

**Figure 2:**
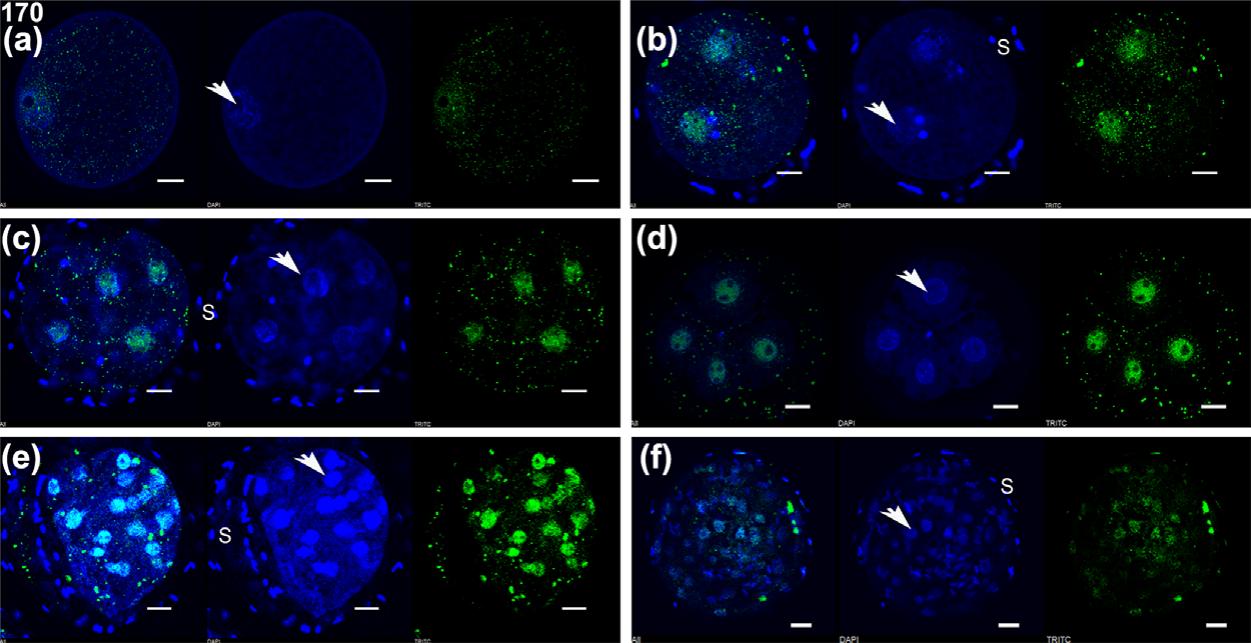
Localization of BAF170 in porcine oocytes and embryos produced in vitro or in vivo. (a) Germinal vesicle‐stage oocyte. (b) Pronuclear embryo produced in vitro. (c) 4‐cell embryo produced in vitro. (d) 4‐cell embryo derived in vivo. (e) Blastocyst‐stage embryo produced in vitro. (f) Blastocyst‐stage embryo derived in vivo. Each panel shows merged images of DNA (blue) and SWI/SNF subunit (pseudo‐colored green) staining (left), or individual DNA (middle) or subunit (right) staining. Arrow, nucleus; S, sperm. Scale bars, 25 μm

**Figure 3:**
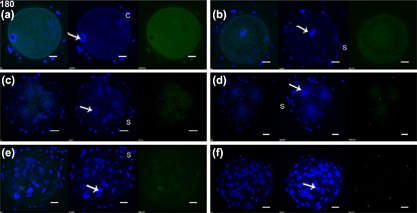
Localization of BAF180 in porcine oocytes and embryos produced in vitro or in vivo. (a) Germinal vesicle‐stage oocyte. (b) Pronuclear embryo produced in vitro. (c) 4‐cell embryo produced in vitro. (d) 4‐cell embryo derived in vivo. (e) Blastocyst‐stage embryo produced in vitro. (f) Blastocyst‐stage embryo derived in vivo. Each panel shows merged images of DNA (blue) and SWI/SNF subunit (pseudo‐colored green) staining (left), or individual DNA (middle) or subunit (right) staining. Arrow, nucleus; C, cumulus cell; S, sperm. Scale bars, 25 μm

**Figure 4:**
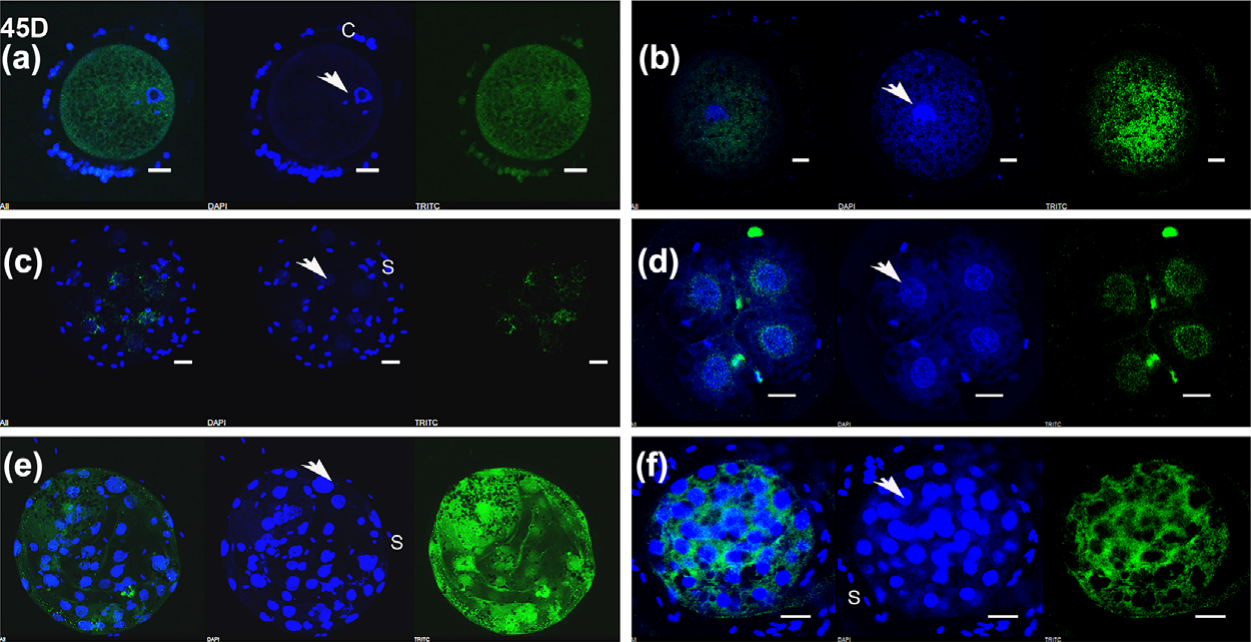
Localization of BAF45D in porcine oocytes and embryos produced in vitro or in vivo. (a) Germinal vesicle‐stage oocyte. (b) Pronuclear embryo produced in vitro. (c) 4‐cell embryo produced in vitro. (d) 4‐cell embryo derived in vivo. (e) Blastocyst‐stage embryo produced in vitro. (f) Blastocyst‐stage embryo derived in vivo. Each panel shows merged images of DNA (blue) and SWI/SNF subunit (pseudo‐colored green) staining (left), or individual DNA (middle) or subunit (right) staining. Arrow, nucleus; C, cumulus cell; S, sperm. Scale bars, 25 μm

**Figure 5:**
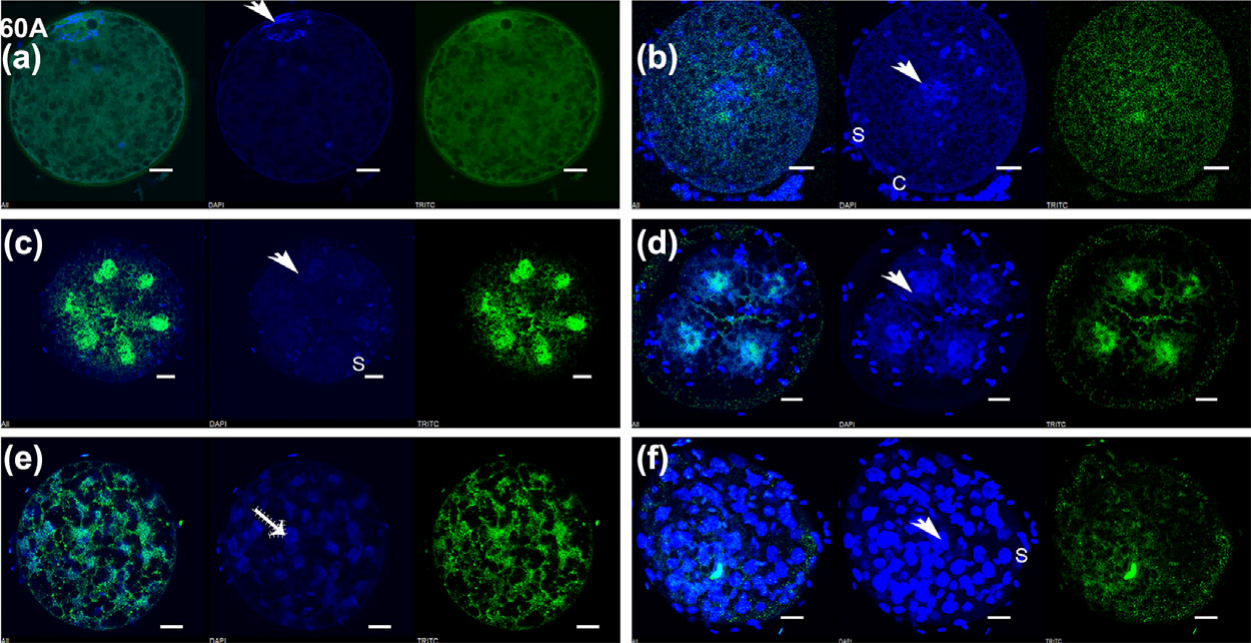
Localization of BAF60A in porcine oocytes and embryos produced in vitro or in vivo. (a) Germinal vesicle‐stage oocyte. (b) Pronuclear embryo produced in vitro. (c) 4‐cell embryo produced in vitro. (d) 4‐cell embryo derived in vivo. (e) Blastocyst‐stage embryo produced in vitro. (f) Blastocyst‐stage embryo derived in vivo. Each panel shows merged images of DNA (blue) and SWI/SNF subunit (pseudo‐colored green) staining (left), or individual DNA (middle) or subunit (right) staining. Arrow, nucleus; C, cumulus cell; S, sperm. Scale bars, 25 μm

**Figure 6:**
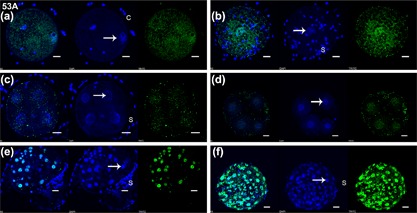
Localization of BAF53A in porcine oocytes and embryos produced in vitro or in vivo. (a) Germinal vesicle‐stage oocyte. (b) Pronuclear embryo produced in vitro. (c) 4‐cell embryo produced in vitro. (d) 4‐cell embryo derived in vivo. (e) Blastocyst‐stage embryo produced in vitro. (f) Blastocyst‐stage embryo derived in vivo. Each panel shows merged images of DNA (blue) and SWI/SNF subunit (pseudo‐colored green) staining (left), or individual DNA (middle) or subunit (right) staining. Arrow, nucleus; C, cumulus cell; S, sperm. Scale bars, 25 μm

**Figure 7:**
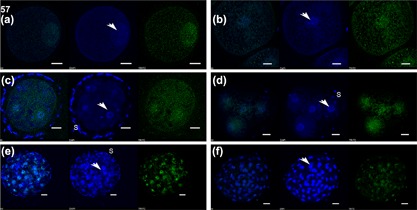
Localization of BAF57 in porcine oocytes and embryos produced in vitro or in vivo. (a) Germinal vesicle‐stage oocyte. (b) Pronuclear embryo produced in vitro. (c) 4‐cell embryo produced in vitro. (d) 4‐cell embryo derived in vivo. (e) Blastocyst‐stage embryo produced in vitro. (f) Blastocyst‐stage embryo derived in vivo. Each panel shows merged images of DNA (blue) and SWI/SNF subunit (pseudo‐colored green) staining (left), or individual DNA (middle) or subunit (right) staining. Arrow, nucleus; S, sperm. Scale bars, 25 μm

**Figure 8:**
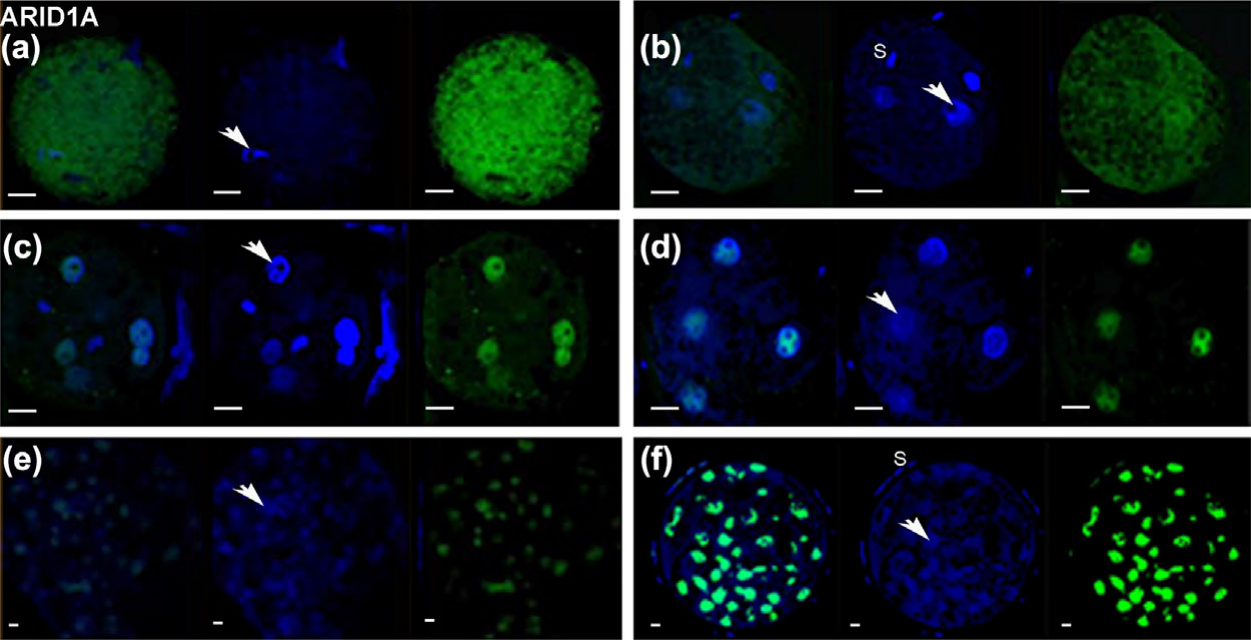
Localization of ARID1A in porcine oocytes and embryos produced in vitro or in vivo. (a) Germinal vesicle‐stage oocyte. (b) Pronuclear embryo produced in vitro. (c) 4‐cell embryo produced in vitro. (d) 4‐cell embryo derived in vivo. (e) Blastocyst‐stage embryo produced in vitro. (f) Blastocyst‐stage embryo derived in vivo. Each panel shows merged images of DNA (blue) and SWI/SNF subunit (green) staining (left), or individual DNA (middle) or subunit (right) staining. Arrow, nucleus; S, sperm. Scale bars, 25 μm (a–d); 10 μm (e,f)

**Figure 9:**
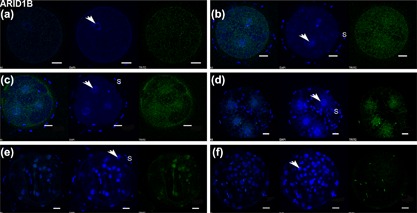
Localization of ARID1B in porcine oocytes and embryos produced in vitro or in vivo. (a) Germinal vesicle‐stage oocyte. (b) Pronuclear embryo produced in vitro. (c) 4‐cell embryo produced in vitro. (d) 4‐cell embryo derived in vivo. (e) Blastocyst‐stage embryo produced in vitro. (f) Blastocyst‐stage embryo derived in vivo. Each panel shows merged images of DNA (blue) and SWI/SNF subunit (pseudo‐colored green) staining (left), or individual DNA (middle) or subunit (right) staining. Arrow, nucleus; S, sperm. Scale bars, 25 μm

**Figure 10:**
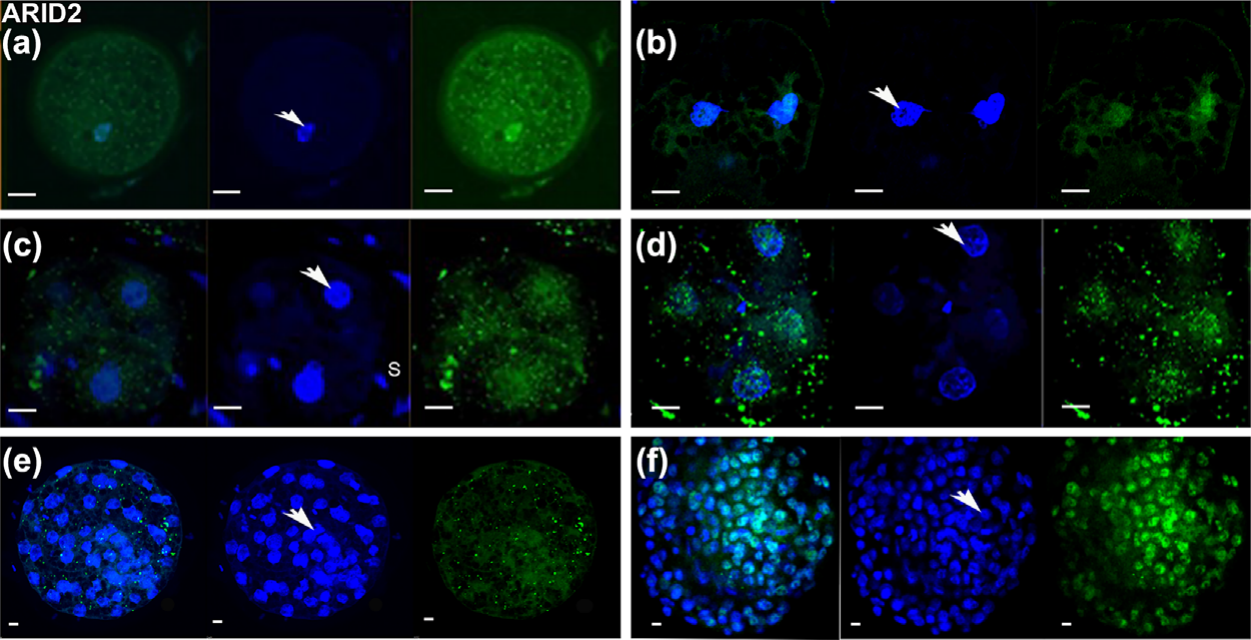
Localization of ARID2 in porcine oocytes and embryos produced in vitro or in vivo. (a) Germinal vesicle‐stage oocyte. (b) Pronuclear embryo produced in vitro. (c) 4‐cell embryo produced in vitro. (d) 4‐cell embryo derived in vivo. (e) Blastocyst‐stage embryo produced in vitro. (f) Blastocyst‐stage embryo derived in vivo. Each panel shows merged images of DNA (blue) and SWI/SNF subunit (green) staining (left), or individual DNA (middle) or subunit (right) staining. Arrow, nucleus; S, sperm. Scale bars, 25 μm (a–d); 10 μm (e,f)

**Figure 11:**
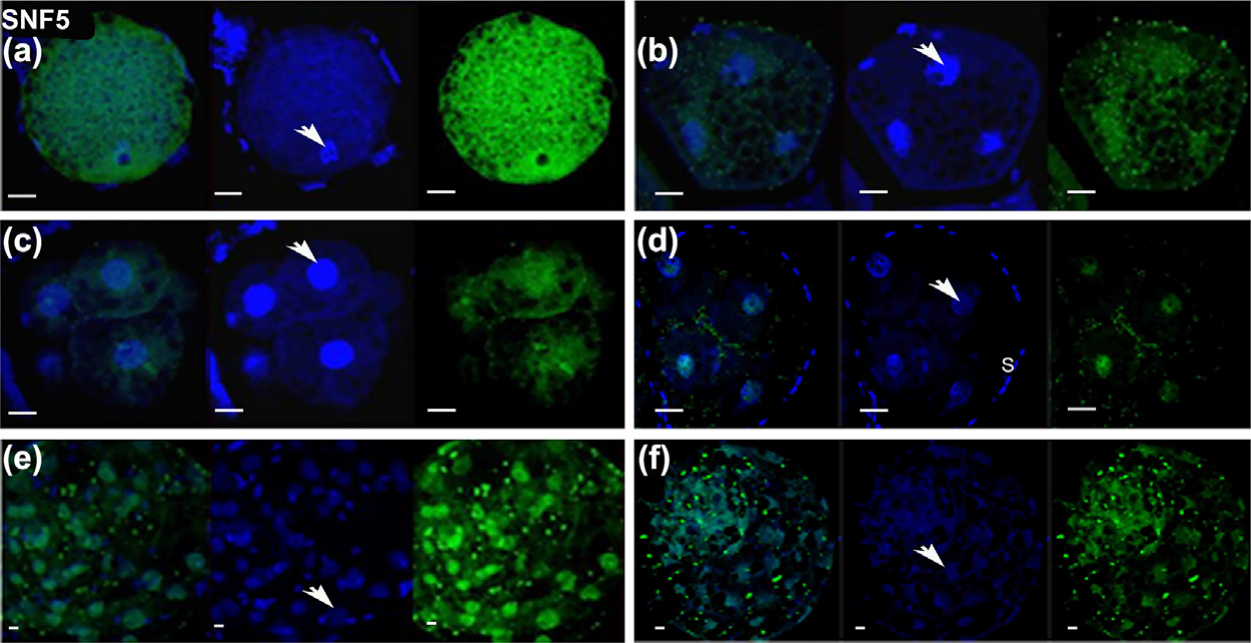
Localization of SNF5 in porcine oocytes and embryos produced in vitro or in vivo. (a) Germinal vesicle‐stage oocyte. (b) Pronuclear embryo produced in vitro. (c) 4‐cell embryo produced in vitro. (d) 4‐cell embryo derived in vivo. (e) Blastocyst‐stage embryo produced in vitro. (f) Blastocyst‐stage embryo derived in vivo. Each panel shows merged images of DNA (blue) and SWI/SNF subunit (green) staining (left), or individual DNA (middle) or subunit (right) staining. Arrow, nucleus; S, sperm. Scale bars, 25 μm (a–d); 10 μm (e,f)

**Figure 12:**
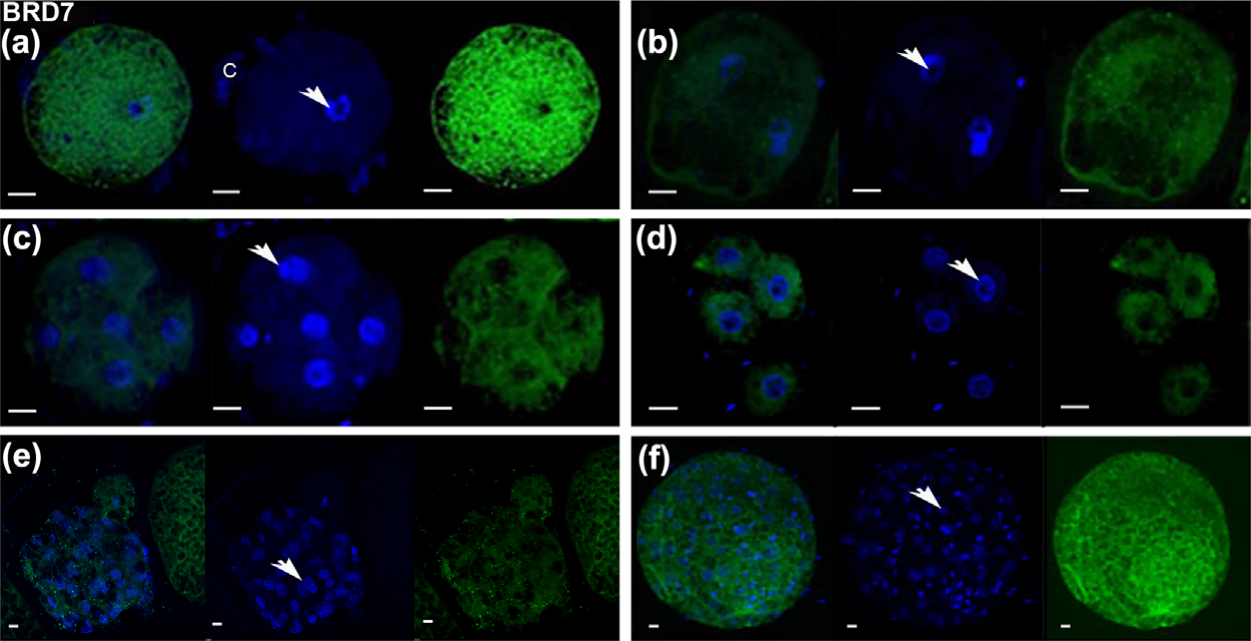
Localization of BRD7 in porcine oocytes and embryos produced in vitro or in vivo. (a) Germinal vesicle‐stage oocyte. (b) Pronuclear embryo produced in vitro. (c) 4‐cell embryo produced in vitro. (d) 4‐cell embryo derived in vivo. (e) Blastocyst‐stage embryo produced in vitro. (f) Blastocyst‐stage embryo derived in vivo. Each panel shows merged images of DNA (blue) and SWI/SNF subunit (green) staining (left), or individual DNA (middle) or subunit (right) staining. Arrow, nucleus. Scale bars, 25 μm (a–d); 10 μm (e,f)

The following results represent data from three independent replicates. Western blot analysis was performed twice to validate the antibodies used in this study (see Supplementary Figure S1). Controls incubated with only secondary antibody exhibited no detectable staining above background levels in any of the oocytes or embryos analyzed. The data presented herein depict representative images of porcine oocytes and embryos probed with antibodies for SWI/SNF subunits, and counterstained with Hoechst to identify the nuclei (Figures 1, 2, 3, 4, 5, 6, 7, 8, 9, 10, 11, 12). A summarized comparison of localization patterns of the 12 SWI/SNF subunits between in vitro‐produced and in vivo‐derived embryos during the course of the first week of development is presented in Table 1. The descriptive localization of each subunit as well as the frequency of each pattern follows.

**Table 1:** Intracellular localization of SWI/SNF factors in porcine oocytes/embryos

Stage	Germinal vesicle	Pronuclear	4‐cell		Blastocyst	
Source	In vitro	In vitro	In vitro	In vitro	In vitro	In vitro
BAF155	N (27/29) Neg (2/29)	N (9/11) Neg (2/11)	N (26/26)	N (6/6)	N* (12/28) C (12/28)	Neg (4/28) N (6/6)
BAF170	N/PeriN (26/33) Neg (7/33)	N (25/25)	N (27/27)	N (5/7) Neg (2/7)	N* (31/34)Neg (3/34)	N (4/6) Neg (2/6)
BAF180	C(18/28)—weak Neg (10/28)	Neg (24/25) N (1/25)—weak	Neg(27/28) N(1/28)—weak	Neg (7/7)	Neg (23/26) N (3/26)—weak	Neg (6/6)
BAF45D	periN (26/27)—weak N/C (1/27)	N/C(13/24)—weak C(8/24)—weak N (3/24)	N/C (9/24) N* (8/24)C(7/24)	N/periN (5/7) C (1/7)½ N, ½ C (1/7)	C (15/27) Neg (12/27)	C(3/7)Neg (4/7)
BAF60A	N (15/26) Neg (11/26)	Neg (12/18), N (6/18)—weak	N (16/27) Neg (11/27)	N (4/4)	N* (10/27)—weak Neg (17/27)	C (3/6)N* (2/6)Neg (1/6)
BAF53A	N (28/33) Neg (5/33)	N (21/25) Neg (4/25)	N/periN (17/23)‐weak Neg (6/23)	N/periN (7/7)	N* (34/34)	N (6/6)
BAF57	N (28/30) Neg (2/30)	N/perN (8/14) Neg (6/14)	N/periN (20/22) Neg (2/22)	N/periN (6/6)	N (25/25)	N (6/6)
ARID1A	N/C (14/14)	N/C (13/13)	N (14/14)	N/periN (6/6)	N (15/15)	N (5/5)
ARID1B	Neg (21/29) N (8/29)—weak	Neg (11/16) N (5/16)—weak	Neg (23/26) periN (3/26)	Neg (6/6)	N*(19/25)Neg (6/25)	N* (1/7) Neg(6/7)
ARID2	N/C (13/18) Neg (5/18)	N (9/13)—weak N/C (4/13)	N (10/14) Neg (4/14)	N (6/6)	N (13/13)	N (4/4)
SNF5	N/C (11/12) Neg (1/12)	N/periN (12/13) C (1/13)—weak	N (11/11)	N (6/6)	N (12/14) Neg (1/14) PeriN (1/14)	N (5/5)
BRD7	C (6/11)—weak N (5/11)—weak	N (6/10)—weak N/C (4/10)—weak	C (11/12) Neg (1/12)	C (11/12) Neg (1/12)	C* (14/17) Neg (3/17)	C* (3/5) Neg (2/5)

C, predominantly cytoplasmic localization; N, predominantly nuclear localization; Neg, no detectable or very weak staining, without preference to either nucleus or cytoplasm; PeriN, perinuclear.

Numbers in parentheses indicate the phenotype frequency (n/total assessed).

* Not all blastomeres were positive.

### 2.1 BAF‐155

Strong nuclear localization of BAF155 was observed in germinal vesicle‐stage oocytes (27/29) and in pronuclear (9/11) and 4‐cell embryos (26/26) produced by in vitro fertilization. Fewer in vitro‐produced blastocyst‐stage embryos exhibited nuclear staining (12/28), and the immunofluorescence signal appeared weaker in these late‐stage embryos; in addition, 4/28 blastocyst‐stage embryos showed no detectable staining, and 12/28 blastocyst‐stage embryos showed only cytoplasmic staining. All in vivo‐derived 4‐cell embryos (6/6) and blastocyst‐stage embryos (6/6) showed strong nuclear staining (Table 1 and Figure 1).

### 2.2 BAF170

The majority of germinal vesicle‐stage oocytes presented clear staining for BAF170 in the nucleus and perinuclear area (26/33), while 7 of 33 germinal vesicle‐stage oocytes showed no detectable staining. BAF170 staining was predominantly nuclear in in vitro‐produced pronuclear (25/25), 4‐cell (27/27), and blastocyst‐stage (31/34) embryos. BAF170 staining was also nuclear in in vivo‐derived 4‐cell (5/7) and blastocyst‐stage (4/6) embryos (Table 1 and Figure 2).

### 2.3 BAF180

BAF180 (also known as PBRM1 [Polybromo protein 1]) is a signature subunit for biochemically‐defined polybromo BAF‐complexes. BAF180 was weakly detected in the cytoplasm of germinal vesicle‐stage oocytes (18/28). BAF180 was not detectable in the majority of in vitro‐produced embryos (24 of 25 pronuclear; 27 of 28 4‐cell; and 23 of 26 blastocyst‐stage embryos). BAF180 was also not detectable in in vivo‐derived embryos at either the 4‐cell (7/7) or blastocyst (6/6) stages (Table 1 and Figure 3).

### 2.4 BAF45D

BAF45D exhibited weak perinuclear localization in germinal vesicle‐stage oocytes (26/27). A varied staining pattern was observed in porcine embryos. In vitro‐produced 4‐cell embryos possessed a ubiquitous staining pattern evenly distributed throughout the nucleus and cytoplasm (9/24), some 4‐cell embryos showed weak BAF45D nuclear localization (8/24) whereas others showed a predominant cytoplasmic localization (7/24). The majority of in vivo‐derived 4‐cell embryos displayed nuclear and perinuclear staining (5/7), while others showed predominant cytoplasmic localization (1/7) or a varied pattern of localization in which two blastomeres possessed cytoplasmic and two blastomeres possessed nuclear localization of BAF45D (1/7). The majority of in vitro‐produced blastocyst‐stage embryos possessed cytoplasmic BAF45D (15/27), although staining was not detectable in the remaining blastocyst‐stage embryos (12/27). The distribution of BAF45D staining was mixed in in vivo‐derived blastocyst‐stage embryos, with three of seven presenting cytoplasmic staining, while staining was not detectable in four of seven embryos (Table 1 and Figure 4).

### 2.5 BAF60A

BAF60A adopted nuclear localization in germinal vesicle‐stage oocytes (15/26), whereas no staining or only weak, ubiquitous staining in both the nucleus and cytoplasm was detected in 11/26 germinal vesicle‐stage oocytes. BAF60A staining was not detectable in the majority of in vitro‐produced pronuclear embryos (12/18), while weak nuclear staining was observed in the remaining in vitro‐produced pronuclear embryos (6/18). BAF60A adopted clear nuclear localization in the majority of in vitro‐produced 4‐cell embryos (16/27). All in vivo‐derived 4‐cell embryos also displayed clear nuclear localization (4/4). Conversely, a varied staining pattern was observed in blastocyst‐stage embryos. BAF60A was not detectable in the majority of in vitro‐produced blastocyst stage embryos (17/27), although weak nuclear staining was detected in discrete blastomeres of some blastocyst‐stage embryos (10/27). In vivo‐derived blastocyst‐stage embryos presented three staining patterns: (i) weak nuclear staining in discrete blastomeres (2/6); (ii) exclusive cytoplasmic staining (3/6); or (iii) no detectable staining in any blastomeres (1/6) (Table 1 and Figure 5).

### 2.6 BAF53A

BAF53A showed predominantly nuclear localization in germinal vesicle‐stage oocytes (28/33). The majority of in vitro‐produced, pronuclear embryos (21/25) showed predominantly nuclear localization. A similar nuclear staining pattern was observed in 4‐cell, in vitro‐produced (17/23) and in in vivo‐derived embryos (7/7). All blastocyst‐stage embryos (34 of 34 in vitro‐produced; 6 of 6 in vivo‐derived) possessed nuclear BAF53A localization (Table 1 and Figure 6).

### 2.7 BAF57

BAF57 was detected in the nuclei of the majority of germinal vesicle‐stage oocytes (28/30). BAF57 was predominantly nuclear in pronuclear (8/14), 4‐cell (20/22), and blastocyst‐stage (25/25) embryos produced in vitro. Localization of BAF57 in in vivo‐derived 4‐cell embryos was perinuclear (2/6) or ubiquitously distributed throughout cytoplasm and nucleus (4/6). All in vivo‐derived blastocyst‐stage embryos (6/6) showed nuclear localization (Table 1 and Figure 7).

### 2.8 ARID1A

ARID1A was evenly distributed between nuclear and cytoplasmic compartments in germinal vesicle‐stage oocytes (14/14) and pronuclear embryos (13/13). ARID1A exhibited strong nuclear localization in 4‐cell embryos produced in vitro (14/14). The majority of in vivo‐derived 4‐cell embryos showed weak nuclear and perinuclear localization (6/6). Both in vitro‐produced and in vivo‐derived blastocysts had predominantly nuclear localization (15/15 and 5/5, respectively) (Table 1 and Figure 8).

### 2.9 ARID1B

ARID1B was not detected in most germinal vesicle‐stage oocytes (21/29) or in in vitro‐ produced pronuclear (11/16) and 4‐cell embryos (23/26). ARID1B was also not detectable in in vivo‐derived 4‐cell embryos (6/6). Most in vitro‐produced blastocyst‐stage embryos (19/25) showed nuclear localization of ARID1B, whereas nuclear localization was detected in only one in vivo‐derived blastocyst‐stage embryo (1/7) (Table 1 and Figure 9).

### 2.10 ARID2

ARID2 was detected in both nuclear and cytoplasmic compartments. It was tightly associated with chromatin in germinal vesicle‐stage oocytes (13/18); had weak nuclear localization in pronuclear embryos (9/13); and localized predominately in the nuclei of 4‐cell and blastocyst‐stage embryos from in vitro production (10/14 and 13/13, respectively) and in vivo derivation (6/6 and 4/4, respectively) (Table 1 and Figure 10).

### 2.11 SNF5

SNF5 was detected in both nuclear and cytoplasmic compartments in germinal vesicle‐stage oocytes (11/12), and had weak perinuclear and nuclear localization in pronuclear embryos (12/13). SNF5 adopted a clear nuclear localization in 4‐cell and blastocyst stages in both in vitro‐produced (11/11 and 12/14, respectively) and in vivo‐derived embryos (6/6 and 5/5, respectively) (Table 1 and Figure 11).

### 2.12 BRD7

The distribution of BRD7 was variable in germinal vesicle‐stage oocytes. While half of the germinal vesicle‐stage oocytes appeared to have cytoplasmic enrichment of BRD7 (6/11), this factor adopted nuclear localization in the other half (5/11). Localization of BRD7 was also variable at the pronuclear stage. Some pronuclear embryos accumulated BRD7 in their nuclei (6/10), whereas others possessed an even distribution between the nucleus and cytoplasm (4/10). BRD7 adopted cytoplasmic localization at the 4‐cell stage in both in vitro‐produced (11/12) and in vivo‐derived (5/6) embryos. At the blastocyst stage, both in vitro produced (14/17) and in vivo‐derived embryos (3/5) showed cytoplasmic BRD7 localization, although some nuclei stained positive for BRD7. The remaining blastocyst‐stage embryos showed no detectable BRD7 staining (3 of 17 and 2 of 15 in vitro‐produced and in vivo‐derived, blastocyst‐stage embryos, respectively) (Table 1 and Figure 12).

## 3 DISCUSSION

The pig embryo exhibits similar timing of zygotic genome activation and blastocyst formation, as well as a similar size, compared to human embryos, making it an excellent non‐primate animal model for early embryo development in humans. The maternal‐to‐zygotic genome transition occurs at the 4‐cell stage in the pig, at which point the zygotic genome becomes activated. The first cellular differentiation event takes place at the blastocyst stage, with formation of the inner cell mass and trophectoderm surrounding the blastocoel. The inner cell mass will give rise to the embryo proper, although these cells exist in an undifferentiated, pluripotent state. On the other hand, trophectoderm cells will contribute only to extra‐embryonic tissues and have undergone differentiation. Here, we analyzed the intracellular localization of 12 SWI/SNF subunits in porcine germinal vesicle‐stage oocytes, which are transcriptionally inactive, and embryos, produced in vitro and in vivo, to understand how they might contribute to these key stages of embryonic genome usage.

In SWI/SNF complexes, the scaffolding subunits BAF155 and BAF170 are present as heterodimers or homodimers (Chen & Archer, 2005; Wang et al., 1996). Wang et al. (2016) found that BAF155 and BAF170 are present within the same remodeling complex in human tissues, at equimolar ratios. Similar to data in mouse embryonic stem cells, we found strong expression of BAF155 in early cleavage‐stage embryos that, in in vitro‐produced embryos, decreased upon differentiation at the blastocyst stage. Unlike data obtained in embryonic stem cells in mice, but similar to findings in human embryonic stem cells (Zhang et al., 2014), BAF170 was detected in most embryos at all stages analyzed. Interestingly, in vitro‐produced blastocysts showed a stronger preference for BAF170 compared to in vivo‐derived blastocysts. This is especially surprising since in vitro‐produced embryos exhibit a slower rate of development (fewer cells and less‐prominent inner cell mass) after in vitro culture compared to in vivo development (reviewed by Lazzari et al., 2010).

The quantity of BAF155 and BAF170 in human cells determines the abundance of BAF57 (Chen & Archer, 2005). In murine embryonic stem cells, knockdown of BAF155 attenuated BAF57 and vice versa (Schaniel et al., 2009). BAF57 is found only in higher eukaryotes, and is a key subunit that facilitates interactions between SWI/SNF complexes and transcription factors (Lomelí & Castillo‐Robles, 2016). With the exception of some pronuclear embryos, BAF57 was detectable in most oocytes and embryos throughout preimplantation development—which is in accordance with reports that BAF57 is omnipresent in all mammalian assemblies (Lomelí & Castillo‐Robles, 2016).

esBAF (SWI/SNF complexes found in embryonic stem cells in mice) is essential for embryonic stem cell self‐renewal and pluripotency. esBAF complexes were defined by the presence of BRG1, BAF155, ARID1A, and BAF60A, as well as the absence of BRM, BAF170, ARID1B, and BAF60C (Ho et al., 2009). Alajem et al. (2015) found both BAF155 and BAF60A to be highly abundant in pluripotent embryonic stem cells, while Takabeyashi et al. (2013) reported high expression of BAF155, BRG1, and BAF53A in murine embryonic stem cells, with their expression decreasing during differentiation. Indeed, knockdown of *Baf155* prevented the down‐regulation of self‐renewal genes, even in the absence of *Pou5f1*, indicating that BAF155‐mediated changes to chromatin structure are the driving force for differentiation of murine embryonic stem cells (Schaniel et al., 2009). Similarly, although knockout of *Baf60a* was tolerated in murine embryonic stem cells, these affected cells died when stimulated to differentiate, pointing to its essential function during stem cell differentiation (Alajem et al., 2015). We found that BAF60A is present in only some in vitro‐produced embryos, and is at low abundance in pronuclear and blastocyst‐stage embryos. In vivo‐derived embryos showed clear nuclear staining at the 4‐cell stage, whereas localization and intensity of the signal was highly varied at the blastocyst stage.

Two additional subunits highlighted for their contribution to cell survival during the process of embryonic stem cell differentiation are SNF5 and BAF53A. SNF5, a core subunit of SWI/SNF chromatin remodeling complexes, is critical for cell survival during the transition from pluripotency to differentiation in murine embryonic stem cells because it controls POU5F1 levels (You et al., 2013). Consistent with this function, we detected SNF5 in the nuclei of the majority of porcine embryos analyzed. In germinal vesicle‐stage oocytes, however, the localization of SNF5 was evenly distributed between the cytoplasm and nucleus. BAF53A is present in both human and murine embryonic stem cells (Lu et al., 2015; Zhang et al., 2014). Additionally in mice, BAF53A repressed differentiation into primitive endoderm (Lu et al., 2015). We detected the presence of BAF53A in a vast majority of porcine oocytes and embryos at all stages analyzed.

Three mutually exclusive members of ARIDs are found within SWI/SNF complexes: ARID1A, ARID1B, and ARID2. In mice, ARID1A is abundant in embryonic stem cells as well as early embryos. The ablation of ARID1A in mice caused developmental arrest around embryonic Day 6.5, and failure to develop a mesodermal layer (Gao et al., 2008). ARID1A was ubiquitously expressed in all regions and stages of early embryos, whereas ARID1B was barely detectable in early embryos, with first detection at the 8‐cell stage in mice (Flores‐Alcantar, Gonzalez‐Sandoval, Escalante‐Alcalde, & Lomelí, 2011). Although murine embryonic stem cells could be established from *Arid1b* ^−/−^ blastocyst‐stage embryos, these cells possessed slower proliferation and an abnormal cell cycle, as well as a lower expression of pluripotency markers and accelerated differentiation in *Arid1b* ^−/−^ versus *Arid1b* ^+/+^ embryonic stem cells (Yan et al., 2008). Similar to data in mice, we found porcine ARID1A in all stages of preimplantation embryos from both in vitro‐ and in vivo‐derived conditions. Conversely, ARID1B was absent in most oocytes and pronuclear and 4‐cell embryos, but was subsequently detected in most in vitro‐produced, blastocyst‐stage embryos—yet, only one of seven in vivo‐derived blastocyst‐stage embryo possessed detectable ARID1B. Such discrepancy in the abundance of ARID1B indicates a potentially altered epigenetic state and abnormal timing of differentiation between blastocyst‐stage embryos produced in vitro versus in vivo.

ARID2, together with BAF180 and BRD7, represent signature subunits for the biochemically defined polybromo BAF complex, a subset of mammalian SWI/SNF chromatin remodeling complexes. This complex is critical in mouse embryo development; indeed *Baf180*‐null mice displayed embryonic lethality (Xu et al., 2012). Our data reveal distinct localizations and abundances of BAF180, ARID2, and BRD7. Very weak or no signal was observed for BAF180, indicating that it is present in low abundance in early porcine embryos or that the BAF180 antigens recognized by this antibody may be masked in whole‐mount immunocytochemical staining due to higher‐order chromatin structure or associations made between BAF180 and other SWI/SNF subunits; an alternative primary antibody for BAF180 could help obtain clearer, brighter images. Additionally—and in contrast to our prediction that members of the polybromo BAF complex would show similar staining patterns—we detected differences between ARID2, which was predominantly nuclear in all analyzed stages of embryo development, and BRD7, which was clearly cytoplasmic at 4‐cell and blastocyst stages of both in vitro‐produced and in vivo‐derived embryos. We interpret these findings to indicate that the classic murine polybromo BAF complexes do not exist in early porcine embryos. Future immunoprecipitation analyses might reveal new combinations of subunits of SWI/SNF complexes in early embryos in the pig. For example, ARID1A/ARID1B and BAF180 can also co‐exist in a subset of SWI/SNF complexes, as revealed in HeLa cells (Ryme et al., 2009).

The majority of the work presented here involved the interpretation of indirect immunofluorescence assays. While the commercially available antibodies used in this series of experiments were not designed to recognize porcine orthologs, a high degree of sequence identity between porcine, murine, and human orthologs suggested the antibodies we chose would detect the porcine orthologs. Immunoreactive bands of equal size were indeed detected in lysates from porcine tissues and human HeLa cells (Supplemental Figure S1); the porcine protein extracts sometimes revealed much clearer banding than protein isolated from HeLa cells (e.g., ARID1A and BAF170). Yet, other antibodies (e.g. against BAF53A, BAF57, and BAF180) exhibited weak immunoreactivity, such that very faint or no bands were detected in porcine tissues. Although this may indicate that the antibody does not recognize the respective porcine orthologs, the absence of non‐specific bands suggested that the equal‐sized band detected is the correct porcine ortholog and that non‐specific binding by immunocytochemical analysis is unlikely. Instead, the porcine tissues examined by immunoblot may not express these particular subunits, or these tissues may possess subunits at a quantity that is below the detection threshold of our assay. One particular example to highlight is BRD7, which was detected as an immunoreactive band of ∼37 kDa in HeLa cells whereas the same antibody detected a single band of ∼70 kDa in porcine tissue. These differing sizes may reflect changes in post‐translational modifications, or degradation of endogenous BRD7 (the predicted mass is 74 kDa). In any case, the absence of non‐specific staining strongly suggests one dominant antigen is recognized by the antibody.

Together, our data indicate that: (i) distinct differences between mouse and pig embryos, as well as differences between embryonic stem cells and early embryos, exist in regards to SWI/SNF subunit use and expression and (ii) the timing of differentiation during in vitro culturing of embryos might not be appropriately synchronized with the expression of certain SWI/SNF subunits, which is reflected by the observed range of SWI/SNF complex subunits present at particular stages of development. Such asynchrony could perturb the epigenetic state of the embryo, potentially resulting in long‐term effects that impact both survival and health of the embryos and subsequent offspring. The variance in localization patterns was more prominent for the in vitro‐produced embryos, suggesting that they are more likely to exhibit asynchronous epigenetic development. Of note, the blastocyst‐stage embryos (both in vitro‐produced and in vivo‐derived) analyzed did not show distinct staining patterns between the inner cell mass and trophectoderm, although we did not explicitly examine the differential intracellular localization of SWI/SNF subunits in these two lineages. Future experiments will focus on SWI/SNF complex distribution in peri‐implantation embryos to determine if embryos are capable of correcting differences in localization and abundance of the various SWI/SNF subunits as well as if these observed differences are carried on during the course of development.

## 4 MATERIALS AND METHODS

### 4.1 Oocyte collection and in vitro production of porcine embryos

Ovaries were obtained from a local slaughterhouse, and transported to the laboratory in an insulated container. Follicular fluid was collected by manual aspiration of antral ovarian follicles. Cumulus‐oocyte complexes were recovered from follicular fluid, and matured at 39°C and 5% CO_2_ in air with 100% humidity, in TIssue culture medium 199 (TCM199) supplemented with 0.14% polyvinyl alcohol, 10 ng/ml Epithelial growth factor, 0.57 mM l‐cysteine, 0.5 IU/ml porcine Follicle‐stimulating hormone, and 0.5 IU/ml ovine Luteinzing hormone, (Abeydeera, Wang, Prather, & Day, 1998). After 44 hr of maturation in vitro, matured oocytes were denuded in 0.1% hyaluronidase in Hepes‐buffered medium containing 0.01% polyvinyl alcohol, and subsequently fertilized with fresh, extended boar semen, according to an established protocol (Abeydeera et al., 1998). Presumptive zygotes were then cultured at 39°C, 5% CO_2_, and 100% humidity in porcine zygote medium 3 (PZM3), supplemented with 3 mg/ml bovine serum albumin (Yoshioka, Suzuki, Tanaka, Anas, & Iwamura, 2002). Presumptive pronuclear oocytes and 4‐cell and blastocyst‐stage embryos were recovered at 20 hr, 48 hr, and 6 days after gamete mixing, respectively.

### 4.2 Collection of in vivo‐derived embryos

Gilts from the Animal Sciences Research and Education Center at Purdue University were inseminated and slaughtered 2–5 days after the onset of estrus to obtain 4‐cell and blastocyst‐stage embryos (Purdue Animal Care and Use Committee protocol 31311000982). Reproductive tracts were removed, and the oviducts/uteri were flushed with Hepes‐buffered medium. Developmental stages and numbers of embryos were recorded, and embryos from each animal were considered as one biological replicate.

### 4.3 Immunocytochemistry

All primary antibodies were obtained from Abcam (Cambridge, MA). Tetramethylrhodamine isothiocyanate (TRITC) and fluorescein isothiocyanate (FITC)‐conjugated secondary antibodies were obtained from Sigma–Aldrich (St. Louis, MO). We analyzed the presence and localization of individual BAF subunits using antibodies to: BAF155 (cat# ab172638), BAF170 (cat# ab84453), SNF5 (cat# 12167) BAF180 (cat# ab196022), BAF45D (cat# ab134942), BAF60A (cat# ab83208), BAF53A (cat# ab84486), BAF57 (cat# ab137081), ARID1A (cat# ab182560), ARID 1B (cat# ab69571), ARID2 (cat# ab113283), and BRD7 (cat# ab56036). Each antibody was tested by Western blot to confirm the detection of proteins of the correct size (Supplemental Figure S1).

Oocytes and embryos were fixed for 90 min at 4°C in 3.7% paraformaldehyde in phosphate‐buffered saline (PBS). After three washes in phosphate‐buffered saline (PBS) supplemented with 0.1% Tween‐20 (PBST), permeabilization was achieved by incubation for 1 hr in PBS containing 1% TritonX‐100, followed by blocking for 12–18 hr at 4°C using blocking solution (0.1 M glycine, 1% goat serum, 0.01% Triton X‐100, 1% powdered nonfat dry milk, 0.5% BSA, 0.02% sodium azide in PBS) (Prather & Rickords, 1992). Oocytes and embryos were incubated overnight at 4°C with respective primary antibodies at dilutions of 1:100 (anti‐BAF180), 1:250 (anti‐BAF45D, anti‐BAF60A), or 1:500 (anti‐BAF155, anti‐BAF170, anti‐SNF5, anti‐BAF53A, anti‐BAF57, anti‐ARID1A, anti‐ARID 1B, anti‐ARID2, anti‐BRD7). After washing extensively three times in PBST, incubation with secondary antibodies (1:500 dilution) was performed at 4°C for 5 hr (TRITC‐conjugated goat anti‐rabbit IgG) or overnight (FITC‐conjugated goat anti‐rabbit IgG). Controls were treated accordingly, with the exception that incubation in primary antibody was omitted. All samples were subsequently stained with Hoechst 33342 (5 μg/ml) for 1 hr, washed extensively with PBST, and mounted on slides in Vectashield solution. Samples were examined using an inverted Nikon A1R_MP multi‐photon confocal microscope, (Nikon Instruments Inc., Melville, NY) using de‐scanned detectors and laser lines at 408 nm (Hoechst), 488 nm (FITC), and 561 nm (TRITC).

## Supporting information

Additional Supporting Information may be found online in the supporting information tab for this article.

**Figure S1**. Validation of antibody specificity.

Supporting Legends S1.

## References

[mrd22922-bib-0001] Abeydeera, L. R. , Wang, W. H. , Prather, R. S. , & Day, B. N. (1998). Maturation in vitro of pig oocytes in protein‐free culture media: Fertilization und subsequent embryo development in vitro. Biology of Reproduction, 58, 1316–1320. 9603270 10.1095/biolreprod58.5.1316

[mrd22922-bib-0002] Alajem, A. , Biran, A. , Harikumar, A. , Sailaja, B. S. , Aaronson, Y. , Livyatan, I. , … Meshorer, E. (2015). Differential association of chromatin proteins identifies BAF60a/SMARCD1 as a regulator of embryonic stem cell differentiation. Cell Reports, 10(12), 2019–2031. https://doi.org/10.1016/j.celrep.2015.02.064 25818293 10.1016/j.celrep.2015.02.064

[mrd22922-bib-0003] Chen, J. , & Archer, T. K. (2005). Regulating SWI/SNF subunit levels via protein‐protein interactions and proteasomal degradation: BAF155 and BAF170 limit expression of BAF57. Molecular and Cellular Biology, 25(20), 9016–9027. 16199878 10.1128/MCB.25.20.9016-9027.2005 PMC1265786

[mrd22922-bib-0004] de la Serna, I. L. , Ohkawa, Y. , & Imbalzano, A. N. (2006). Chromatin remodelling in mammalian differentiation: Lessons from ATP‐dependent remodellers. Review. Nature Reviews Genetics, 7(6), 461–473. 10.1038/nrg1882 16708073

[mrd22922-bib-0005] Endo, T. , Imai, A. , Shimaoka, T. , Kano, K. , & Naito, K. (2011). Histone exchange activity and its correlation with histone acetylation status in porcine oocytes. Reproduction, 141(4), 397–405. https://doi.org/10.1530/REP‐10‐0164 21239526 10.1530/REP-10-0164

[mrd22922-bib-0006] Euskirchen, G. , Auerbach, R. K. , & Snyder, M. (2012). SWI/SNF chromatin‐remodeling factors: Multiscale analyses and diverse functions. The Journal of Biological Chemistry, 287(37), 30897–30905. https://doi.org/10.1074/jbc.R111.309302 22952240 10.1074/jbc.R111.309302 PMC3438922

[mrd22922-bib-0007] Flores‐Alcantar, A. , Gonzalez‐Sandoval, A. , Escalante‐Alcalde, D. , & Lomelí, H. (2011). Dynamics of expression of ARID1A and ARID1B subunits in mouse embryos and in cells during the cell cycle. Cell and Tissue Research, 345(1), 137–148. 21647563 10.1007/s00441-011-1182-x

[mrd22922-bib-0008] Gao, X. , Tate, P. , Hu, P. , Tjian, R. , Skarnes, W. C. , & Wang, Z. (2008). ES cell pluripotency and germ‐layer formation require the SWI/SNF chromatin remodeling component BAF250a. Proceedings of the National Academy of Sciences of the United States of America, 105(18), 6656–6661. 18448678 10.1073/pnas.0801802105 PMC2373334

[mrd22922-bib-0009] Hamm, J. , Tessanne, K. , Murphy, C. N. , & Prather, R. S. (2014). Transcriptional regulators TRIM28, SETDB1, and TP53 are aberrantly expressed in porcine embryos produced by in vitro fertilization in comparison to in vivo‐ and somatic‐cell nuclear transfer‐derived embryos. Molecular Reproduction and Development, 81(6), 552–566. https://doi.org/10.1002/mrd.22324 24659575 10.1002/mrd.22324 PMC4235398

[mrd22922-bib-0010] Ho, L. , Ronan, J. L. , Wu, J. , Staahl, B. T. , Chen, L. , Kuo, A. , … Crabtree, G. R. (2009). An embryonic stem cell chromatin remodeling complex, esBAF, is essential for embryonic stem cell self‐renewal and pluripotency. Proceedings of the National Academy of Sciences of the United States of America, 106(13), 5181–5186. https://doi.org/10.1073/pnas.0812889106 19279220 10.1073/pnas.0812889106 PMC2654396

[mrd22922-bib-0011] Kadoch, C. , & Crabtree, G. R. (2015). Mammalian SWI/SNF chromatin remodeling complexes and cancer: Mechanistic insights gained from human genomics. Review. Science Advances, 1(5), e1500447 https://doi.org/10.1126/sciadv.1500447 26601204 10.1126/sciadv.1500447 PMC4640607

[mrd22922-bib-0012] Kadoch, C. , Hargreaves, D. C. , Hodges, C. , Elias, L. , Ho, L. , Ranish, J. , & Crabtree, G. R. (2013). Proteomic and bioinformatic analysis of mammalian SWI/SNF complexes identifies extensive roles in human malignancy. Nature Genetics, 45(6), 592–601. https://doi.org/10.1038/ng.2628 23644491 10.1038/ng.2628 PMC3667980

[mrd22922-bib-0013] Kidder, B. L. , Plamer, S. , & Knott, J. G. (2009). SWI/SNF‐Brg1 regulates self‐renewal and occupies core pluripotency‐related genes in embryonic stem cells. Stemm Cells, 27(2), 317–328. 10.1634/stemcells.2008-0710 19056910

[mrd22922-bib-0014] Kim J. K. , Huh, S. O. , Choi, H. , … Seong R. H. (2001). Srg3, a mouse homolog of yeast SWI3, is essential for early embryogenesis and involved in brain development. Molecular Cell Biology, 21(22), 7787–95. https://doi.org/10.1128/MCB.21.22.7787–7795.2001 10.1128/MCB.21.22.7787-7795.2001 PMC99948 11604513

[mrd22922-bib-0015] Lazzari, G. , Colleoni, S. , Lagutina, I. , Crotti, G. , Turini, P. , Tessaro I. , … Galli, C. (2010). Short‐term and long‐term effects of embryo culture in the surrogate sheep oviduct versus in vitro culture for different domestic species. Theriogenology, 73(6), 748–757. https://doi.org/10.1016/j.theriogenology.2009.08.001 19726075 10.1016/j.theriogenology.2009.08.001

[mrd22922-bib-0016] Lomelí, H. , & Castillo‐Robles, J. (2016). The developmental and pathogenic roles of BAF57, a special subunit of the BAF chromatin‐remodeling complex. Review. FEBS Letters, 590(11), 1555–1569. https://doi.org/10.1002/1873‐3468.12201 27149204 10.1002/1873-3468.12201

[mrd22922-bib-0017] Lu, W. , Fang, L. , Ouyang, B. , & Songyang, Z. (2015). Actl6a protects embryonic stem cells from differentiating into primitive endoderm. Stem Cells, 33(6), 1782–1793. https://doi.org/10.1002/stem.2000 25802002 10.1002/stem.2000

[mrd22922-bib-0018] Marquez‐Vilendrer, S. B. , Rai, S. K. , Gramling, S. J. , Lu, L. , & Reisman, D. N. (2016). BRG1 and BRM loss selectively impacts RB and P53, respectively: BRG1 and BRM have differential functions in vivo. Oncoscience, 3(11–12), 337–350. https://doi.org/10.18632/oncoscience.333 28105458 10.18632/oncoscience.333 PMC5235922

[mrd22922-bib-0019] Østrup, O. , Olbricht, G. , Østrup, E. , Hyttel, P. , Collas, P. , & Cabot, R. (2013). RNA profiles of porcine embryos during genome activation reveal complex metabolic switch sensitive to in vitro conditions. PLoS ONE, 8(4), e61547 https://doi.org/10.1371/journal.pone.0061547 23637850 10.1371/journal.pone.0061547 PMC3639270

[mrd22922-bib-0020] Panamarova, M. , Cox, A. , Wicher, K. B. , & Zernicka‐Goetz, M. (2016). The BAF chromatin remodeling complex is an epigenetic regulator of lineage specification in the early mouse embryo. Development, 143(8), 1271–1283. http://doi.org/10.1242/dev.131961 26952987 10.1242/dev.131961 PMC4852518

[mrd22922-bib-0021] Park, K. E. , Johnson, C. M. , Magnani, L. , Wang, X. , Biancardi, M. N. , & Cabot, R. A. (2010). Global H3K9 dimethylation status is not affected by transcription, translation, or DNA replication in porcine zygotes. Molecular Reproduction and Development, 77(5), 420–429. https://doi.org/10.1002/mrd.21156 20108327 10.1002/mrd.21156

[mrd22922-bib-0022] Park, K. E. , Johnson, C. M. , Wang, X. , & Cabot, R. A. (2011). Differential developmental requirements for individual histone H3K9 methyltransferases in cleavage‐stage porcine embryos. Reproduction, Fertility, and Development, 23(4), 551–560. https://doi.org/10.1071/RD10280 10.1071/RD10280 21557922

[mrd22922-bib-0023] Park, K. E. , Magnani, L. , & Cabot, R. A. (2009). Differential remodeling of mono‐ and trimethylated H3K27 during porcine embryo development. Molecular Reproduction and Development, 76(11), 1033–1042. https://doi.org/10.1002/mrd.21061 19536841 10.1002/mrd.21061

[mrd22922-bib-0024] Prather, R. S. , & Rickords, L. F. (1992). Developmental regulation of an snRNP core protein epitope during pig embryogenesis and after nuclear transfer for cloning. Molecular Reproduction and Development, 33(2), 119–123. 1384573 10.1002/mrd.1080330202

[mrd22922-bib-0025] Reisman, D. , Glaros, S. , & Thompson, E. A. (2009). The SWI/SNF complex and cancer. Review. Oncogene, 28(14), 1653–1668. https://doi.org/10.1038/onc.2009.4 19234488 10.1038/onc.2009.4

[mrd22922-bib-0026] Ryme, J. , Asp, P. , Böhm, S. , Cavellán, E. , & Farrants, A. K. (2009). Variations in the composition of mammalian SWI/SNF chromatin remodelling complexes. Journal of Cellular Biochemistry, 108(3), 565–576. https://doi.org/10.1002/jcb.22288 19650111 10.1002/jcb.22288

[mrd22922-bib-0027] Schaniel, C. , Ang, Y. S. , Ratnakumar, K. , Carmier, C. , James, T. , Bernstein, E. , … Paddison, P. J. (2009). Smarcc1/Baf155 couples self‐renewal gene repression with changes in chromatin structure in mouse embryonic stem cells. Stem Cells, 27(12), 2979–2991. 19785031 10.1002/stem.223 PMC5978760

[mrd22922-bib-0028] Sun, Q. Y. , Wu, G. M. , Lai, L. , Bonk, A. , Cabot, R. , Park, K. W. , … Schatten, H. (2002). Regulation of mitogen‐activated protein kinase phosphorylation, microtubule organization, chromatin behavior, and cell cycle progression by protein phosphatases during pig oocyte maturation and fertilization in vitro. Biology of Reproduction, 66(3), 580–588. 11870061 10.1095/biolreprod66.3.580

[mrd22922-bib-0029] Takabeyashi S. I. , Lei I. , Ryba T. , Sasaki T. , Dileep V. , Battaglia D. , … Gilbert D. M. (2013). Murine esBAF chromatin remodeling complex subunits BAF250a and Brg1 are necessary to maintain and reprogram pluripotency‐specific replication timing of select replication domains. Epigentics & Chromatin, 6, 42. 10.1186/1756-8935-6-42 PMC3895691 24330833

[mrd22922-bib-0030] Wang, W. , Xue, Y. , Zhou, S. , Kuo, A. , Cairns, B. R. , & Crabtree, G. R. (1996). Diversity and specialization of mammalian SWI/SNF complexes. Genes & Development, 10(17), 2117–2130. 8804307 10.1101/gad.10.17.2117

[mrd22922-bib-0031] Wang, X. , Lee, R. S. , Alver, B. H. , Haswell, J. R. , Wang, S. , Mieczkowski, J. , … Roberts, C. W. (2016). SMARCB1‐mediated SWI/SNF complex function is essential for enhancer regulation. Nature Genetics, 49(2), 289–295. https://doi.org/10.1038/ng.3746 27941797 10.1038/ng.3746 PMC5285474

[mrd22922-bib-0032] Xu, F. , Flowers, S. , & Moran, E. (2012). Essential role of ARID2 protein‐containing SWI/SNF complex in tissue‐specific gene expression. The Journal of Biological Chemistry, 287(7), 5033–5041. https://doi.org/10.1074/jbc.M111.279968 22184115 10.1074/jbc.M111.279968 PMC3281626

[mrd22922-bib-0033] Yan, Z. , Wang, Z. , Sharov, A. A. , Ling, C. , Piao, Q. , Aiba, K. , … Ko, M. S. (2008). BAF250B‐associated SWI/SNF chromatin‐remodeling complex is required to maintain undifferentiated mouse embryonic stem cells. Stem Cells, 26(5), 1155–1165. 18323406 10.1634/stemcells.2007-0846 PMC2409195

[mrd22922-bib-0034] Yoshioka, K. , Suzuki, C. , Tanaka, A. , Anas, I. M. , & Iwamura, S. (2002). Birth of piglets derived from porcine zygotes cultured in a chemically defined medium. Biology of Reproduction, 66(1), 112–119. 11751272 10.1095/biolreprod66.1.112

[mrd22922-bib-0035] You, J. S. , De Carvalho, D. D. , Dai, C. , Liu, M. , Pandiyan, K. , Zhou, X. J. , … Jones, P. A. (2013). SNF5 is an essential executor of epigenetic regulation during differentiation. PLoS Genetics, 9(4), e1003459 https://doi.org/10.1371/journal.pgen.1003459 23637628 10.1371/journal.pgen.1003459 PMC3636213

[mrd22922-bib-0036] Zhang, X. , Li, B. , Li, W. , Ma, L. , Zheng, D. , Li, L. , … Wang, Y. (2014). Transcriptional repression by the BRG1‐SWI/SNF complex affects the pluripotency of human embryonic stem cells. Stem Cell Reports, 3(3), 460–474. https://doi.org/10.1016/j.stemcr.2014.07.004 25241744 10.1016/j.stemcr.2014.07.004 PMC4266000

