# Supplementary figures and images for: Differential expression of key subunits of SWI/SNF chromatin remodeling complexes in porcine embryos derived in vitro or in vivo
Source: Mol Reprod Dev. 2017 Nov 6;84(12):1238–49. doi: 10.1002/mrd.22922 (PMC5760298; doi:10.1002/mrd.22922)

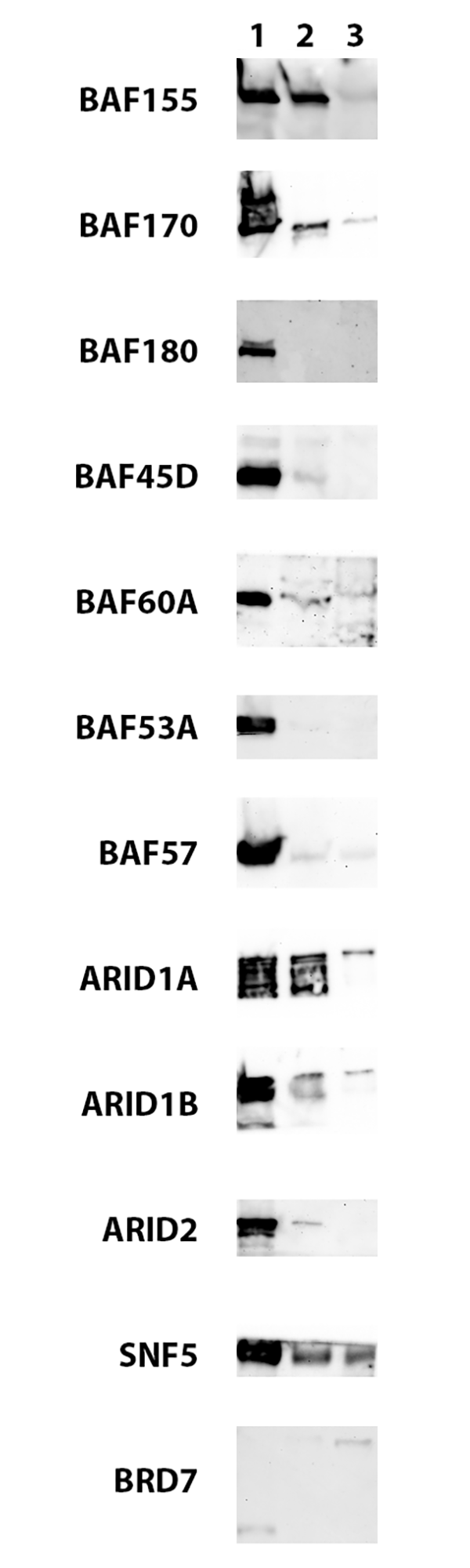

Supplement: Supplementary file 1 — Figure S1. Validation of antibody specificity. [file MRD-84-1238-s001.tif]
