# Supplementary material for: Differential expression of key subunits of SWI/SNF chromatin remodeling complexes in porcine embryos derived in vitro or in vivo
Source: Mol Reprod Dev. 2017 Nov 6;84(12):1238–49. doi: 10.1002/mrd.22922 (PMC5760298; doi:10.1002/mrd.22922)
Supplement: Supplementary file 2 — Supporting Legends S1. [file MRD-84-1238-s002.docx]

**Figure legend**

**Supplementary Figure 1**

Western blot analysis of SWI/SNF subunits in porcine tissues. Every Western blot was performed twice. For BAF155 (123 kDa) and SNF5 (42-45 kDa), Lane 1 contains 40 μg HeLa nuclear protein extract; Lane 2 contains 40 μg of porcine fibroblast cell protein extract; Lane 3 contains 40 µg of porcine liver protein. For BAF170 (162, 170 kDa), BAF180 (193 kDa), BAF45D (44 kDa), BAF60A (58 kDa), BAF53A (53 kDa), BAF57 (47 kDa), ARID1A (165-320 kDa), and ARID1B (170 kDa), Lane 1 contains 40 µg HeLa nuclear protein extract; Lane 2 contains 40 µg of porcine fibroblast cell protein extract; Lane 3 contains 40 µg of a nuclear protein extract from porcine fibroblast cells. For ARID2 (240 kDa) and BRD7 (56, 74 kDa), Lane 1 contains 40 µg HeLa nuclear protein extract; Lane 2 contains 40 µg of porcine fibroblast cell nuclear protein extract; Lane 3 contains protein obtained from 750-1000 germinal vesicld-stage porcine oocytes.

**Methods and Materials**

Porcine fibroblast protein was isolated from confluent cultures of porcine fibroblast derived from a porcine conceptus (Day 40 of gestation), between passages 3-5. Protein was isolated using T-PER reagent (ThermoFisher Scientific), according to the manufacturer’s instructions. Nuclear protein fractions were obtained from porcine fibroblast cells using established methods (Dykhuizen et al., 2013). Briefly, porcine fetal fibroblast cells were pelleted and resuspended in 10 mL of a 4˚C wash buffer containing 50 mM HEPES, 140 mM NaCl, 1 mM EDTA, 10% glycerol, 0.5% NP40, and 0.25% Triton X100 (pH 8.0). Resuspended cells were incubated on ice for 10 minutes, and centrifuged at 1,200 g for 5 minutes. The resuspended cells were then washed in a 4^˚^C wash buffer containing 10 mM Tris, 200 mM NaCl, 1 mM EDTA, and 0.5 mM EGTA (pH 8.0). The cell pellet was resuspended in 4^˚^C lysis buffer containing 0.1% SDS, 1mM EDTA, and 10 mM Tris HCl (pH 8.0). Protein samples were mixed with Laemmli sample buffer, boiled for 5 minutes, and loaded into precast 10% acrylamide gels (Bio-Rad, Hercules, CA). Following electrophoresis, samples were transferred to nitrocellulose membranes with a 0.22-µm pore size. Membranes were blocked for 1 hour at room temperature in 5% bovine serum albumin in Tris-buffered saline containing 0.1% Tween20 (TBST). Membranes were probed with primary antibody at 4˚C for 12-18 hours. Primary antibodies were diluted as follows: BAF155 (1:1000), BAF170 (1:5000), BAF180 (1:10,000), BAF45D (1:10,000), BAF60A (1:750), BAF53A (1:7500), BAF57 (1:5000), ARID1A (1:1000), ARID1B (1:2500), ARID2 (1:1000), SNF5 (1:1000), and BRD7 (1:1000) (see main text for sources). Membranes were washed in TBST and incubated for 1 hour at room temperature with HRP-conjugated secondary antibody (goat anti-rabbit IgG diluted 1:5000) in 5% milk in TBST. After secondary antibody staining, membranes were washed in TBST three times (5 minutes for each wash), incubated with Clarity Max Western ECL Substrate (Bio-Rad) for 5 minutes, and imaged on a FluroChem R (Protein Simple).
